# Effects of Bee Bread (Perga) on Pro-Inflammatory Cytokine Levels and Histopathological Alterations in the Liver and Kidneys of Streptozotocin-Induced Diabetic Rats

**DOI:** 10.3390/biology15050380

**Published:** 2026-02-26

**Authors:** Nur Akman, Turan Yaman, Ahmet Ufuk Kömüroğlu, Meryem Çalışır

**Affiliations:** 1 Department of Midwifery, Faculty of Health Sciences, Van Yüzüncü Yıl University, 65080 Van, Turkey; 2 Department of Pathology, Faculty of Veterinary Medicine, Van Yüzüncü Yıl University, 65080 Van, Turkey; turanyaman@yyu.edu.tr; 3 Vocational School of Health Services, Van Yüzüncü Yıl University, 65080 Van, Turkey; aukomuroglu@yyu.edu.tr (A.U.K.); meryemcalisir@yyu.edu.tr (M.Ç.)

**Keywords:** diabetes mellitus, bee bread, perga, cytokines, kidney, histopathology, inflammation

## Abstract

Diabetes mellitus (DM) is a chronic metabolic disorder characterized by persistent systemic inflammation, which contributes to progressive multi-organ dysfunction, particularly in metabolically active tissues such as the liver and kidneys. Bee bread (Perga), a fermented bee pollen product rich in bioactive compounds, has been reported to exert anti-inflammatory and organ-protective effects; however, its tissue-specific influence on inflammatory responses under diabetic conditions remains incompletely defined. Thirty-two male Wistar Albino rats were randomly assigned to four groups: Control, DM, DM + Perga, and Perga. Diabetes was induced by streptozotocin (STZ; 55 mg/kg, i.p.). Perga was administered orally at a dose of 0.5 g/kg/day for 28 days. Pro-inflammatory cytokine levels (CRP, TNF-α, IL-1β, and IL-6) were quantified in liver and kidney tissues using ELISA. Histopathological alterations were evaluated by hematoxylin and eosin staining. DM significantly increased the IL-1β, IL-6, and CRP levels in hepatic tissue and elevated TNF-α, IL-1β, IL-6, and CRP levels in renal tissue. Perga administration attenuated these inflammatory responses, particularly reducing IL-1β and IL-6 levels in the liver and all measured cytokines in the kidney. Histopathological analyses revealed hepatocyte degeneration and necrosis, sinusoidal dilatation, tubular epithelial degeneration, and glomerular damage in diabetic rats, whereas Perga treatment partially improved hepatic alterations and improved renal structural integrity. These findings indicate that Perga exerts tissue-specific anti-inflammatory and protective effects in experimental diabetes, with a more pronounced impact on renal inflammation than on hepatic responses. Although its effects on hepatic TNF-α and CRP levels were limited, Perga may act as a natural modulator of cytokine-mediated inflammatory processes. Further studies are warranted to elucidate the underlying molecular mechanisms.

## Simple Summary

Diabetes is a chronic disease that leads to high blood sugar and persistent inflammation, which can progressively damage vital organs such as the liver and kidneys. Preventing inflammation-related organ injury is essential to reduce long-term complications. Bee bread, also known as perga, is a natural fermented bee product rich in biologically active compounds with potential anti-inflammatory properties. In this study, we examined whether bee bread could protect the liver and kidneys in rats with experimental diabetes. Diabetes caused significant increases in inflammatory markers and structural damage in both organs. Bee bread treatment markedly reduced inflammation and improved tissue structure in the kidneys. However, although some inflammatory markers decreased in the liver, structural recovery was limited. This difference between the two organs may be related to variations in local cellular signaling and immune regulation mechanisms. Overall, our findings suggest that bee bread exerts organ-specific protective effects, with a stronger benefit in the kidneys than in the liver. These results highlight the importance of understanding tissue-specific responses when evaluating natural products for diabetes-related complications.

## 1. Introduction

Diabetes mellitus (DM) is a chronic metabolic disorder with a rapidly increasing global prevalence, characterized by dysregulation of carbohydrate, lipid, and protein metabolism, leading to multi-organ dysfunction [1]. Among the most affected organs, the liver and kidneys are not only highly metabolically active but also particularly susceptible to diabetic vascular and inflammatory complications [2]. The liver plays a central role in glucose homeostasis; however, DM can induce pathological alterations including abnormal glycogen synthesis, fibrosis, cirrhosis, hepatitis, and acute liver injury [3]. Similarly, diabetic kidney disease may progress silently in Type 1 DM and often develops even before the clinical diagnosis of Type 2 DM, highlighting the vulnerability of renal tissue to hyperglycemia-induced damage [2].

Emerging evidence suggests that low-grade chronic inflammation is intricately linked to DM and its complications, particularly in the liver and kidneys [3,4,5]. Proinflammatory cytokines, including tumor necrosis factor-alpha (TNF-α) and interleukin-1 beta (IL-1β), can disrupt insulin signaling and lipid metabolism, thereby exacerbating insulin resistance [6,7]. C-reactive protein (CRP), a key systemic inflammation marker, has also been correlated with increased risk and progression of DM when elevated [8]. Importantly, therapeutic interventions targeting proinflammatory cytokines have been shown to improve insulin sensitivity, glucose metabolism, and organ protection in diabetic models [9].

Bee bread (Perga) is a naturally fermented product of pollen and honey, enriched by lactic acid bacteria and bee-derived enzymes. Traditionally, it has been used for its antimicrobial, antioxidant, anti-inflammatory, and anticancer properties [10,11]. Perga contains water, proteins, carbohydrates, lipids, inorganic elements, and a wide range of bioactive compounds, including decanoic acid, vitamins B and C, nucleic acids, pantothenic acid, biopterin, acetylcholine, flavonoids, neopterin, and reproductive hormones [12]. Experimental studies have demonstrated that Perga exhibits antioxidant activity [13], inhibits the formation of advanced glycation end-products [12], suppresses carbohydrate-hydrolyzing enzymes [13], enhances insulin sensitivity [14], modulates gut microbiota composition [15], and regulates insulin gene expression and cytokine secretion, collectively contributing to improved glycemic control in diabetic models [16,17]. Previous investigations also indicate that Perga and other bee-derived products attenuate hepatocellular injury and reduce pro-inflammatory cytokines such as TNF-α, IL-1β, and IL-6 in experimental animals [18,19]. Moreover, Perga has been shown to mitigate renal tissue injury by suppressing inflammatory mediators, preserving tubular integrity, and improving renal functional parameters [20,21]. Mechanistically, these protective effects are believed to involve modulation of the nuclear factor kappa B (NF-κB) signaling pathway and downregulation of pro-inflammatory cytokine expression [18,19,20,21].

Despite accumulating experimental evidence supporting the anti-inflammatory and organ-protective properties of Perga and other bee-derived products [18,19,20,21,22,23], most available data derive from metabolic dysfunction, obesity, or toxin-induced injury models rather than diabetes-specific organ inflammation. Comparative evaluation of cytokine modulation in metabolically distinct organs such as the liver and kidneys under diabetic conditions remains limited [22,23]. Recent studies in streptozotocin-induced diabetic models have shown that various natural and pharmacological agents can ameliorate hepatic and renal injury by modulating inflammatory cytokines and oxidative stress pathways [24,25]. However, investigations simultaneously assessing and directly comparing Perga’s effects on organ-specific cytokine regulation in both liver and kidney tissues under diabetic conditions remain limited. Moreover, clinical evidence evaluating Perga as a complementary therapeutic strategy in diabetes-related organ injury remains limited, underscoring the need for translational investigations bridging experimental findings to clinical applications. Given the central roles of the liver and kidneys in glucose homeostasis and their pronounced susceptibility to inflammation-induced injury under diabetic conditions, evaluating Perga’s tissue-specific influence on cytokine expression may provide important insights into its therapeutic potential. Therefore, this study aimed to investigate the effects of Perga on TNF-α, IL-1β, IL-6, and CRP levels in the liver and kidney tissues of streptozotocin-induced diabetic rats. The findings may help clarify the role of Perga as a natural modulator of organ-specific inflammatory responses and contribute to the development of complementary strategies for diabetes management.

## 2. Materials and Methods

### 2.1. Chemicals

Streptozotocin (STZ; Sigma-Aldrich, St. Louis, MO, USA), ketamine (Ketaset, Fort Dodge, IA, USA), and xylazine (Rompun 2%; Bayer, Leverkusen, Germany) were used for experimental procedures. Enzyme-linked immunosorbent assay (ELISA) kits were obtained from Cloud-Clone Corp. (Wuhan, Hubei, China) for CRP (SEA821Ra), TNF-α (SEA133Ra), IL-6 (SEA079Ra), and IL-1β (SEA563Ra) and used according to the manufacturer’s protocols.

### 2.2. Experimental Animals

Thirty-two male Wistar albino rats (170–200 g) were obtained from the Van Yüzüncü Yıl University Experimental Medicine Research and Application Center. Animals were housed under standardized conditions (12 h light/dark cycle, temperature 22 ± 2 °C, humidity 55–60%) in standard plastic cages with ad libitum access to water and a standard pellet diet.

All procedures were approved by the Van Yüzüncü Yıl University Local Ethics Committee for Animal Experiments (Approval No: 2024/12-03; 26 December 2024) and conducted in accordance with institutional guidelines and international ARRIVE recommendations.

### 2.3. Bee Bread (Perga) Preparation and Administration

Fresh Perga was obtained from a local beekeeper, dried at 35 °C for 4 h, and ground into a fine powder. The powder was freshly prepared each day by dissolving 1 mL of distilled water and administered orally via gavage at a dose of 0.5 g/kg body weight/day for 28 consecutive days. The selected dose was based on previous studies demonstrating antioxidant and anti-inflammatory efficacy in diabetic models [22,23].

### 2.4. Experimental Induction of Diabetes

Diabetes was induced in overnight-fasted rats (except those in the Control group) using a single intraperitoneal injection of STZ (55 mg/kg), freshly dissolved in 0.1 M sodium citrate buffer (pH 4.5) [26]. Rats in the Control and Perga-only groups received an equivalent volume of citrate buffer.

Seventy-two hours after STZ administration, blood samples were collected from the tail vein, and fasting blood glucose levels were measured using an Accu-Chek Active glucometer (Roche Diagnostics GmbH, Mannheim, Germany). Rats with glucose levels > 200 mg/dL were considered diabetic and included in the study [1]. To prevent acute hypoglycemia, 10% dextrose solution was provided as drinking water immediately after STZ injection.

### 2.5. Experimental Design and Grouping

Rats were randomly assigned into four experimental groups (*n* = 8 per group) as follows:

- **Group 1 (Control):** No treatment was applied.
- **Group 2 (DM):** Diabetes was induced by a single intraperitoneal injection of STZ (55 mg/kg).
- **Group 3 (DM + Perga):** Diabetes was induced as described above, followed by the oral administration of Perga (0.5 g/kg/day) beginning three days after STZ injection and continued for 28 days.
- **Group 4 (Perga):** Perga (0.5 g/kg/day) was administered orally for 28 days without diabetes induction.

At the end of the 28-day experimental period, all rats were anesthetized, euthanized, and their liver and kidney tissues were collected. The harvested samples were used for biochemical analyses of CRP, TNF-α, IL-1β, and IL-6 using ELISA techniques.

### 2.6. Collection of Liver and Kidney Tissue Samples and Biochemical Analyses

At the end of the experimental period, all rats were euthanized by intraperitoneal administration of ketamine (90 mg/kg) and xylazine (10 mg/kg). Liver and kidney tissues were immediately excised, rinsed with cold physiological saline, and homogenized in 50 mM potassium buffer (pH 7.4). The homogenates were centrifuged at 14,000 rpm for 15 min at 4 °C to obtain the supernatants. The resulting supernatants were stored at −20 °C until biochemical analyses were performed [27].

### 2.7. Determination of CRP, TNF-α, IL-1β, and IL-6 Levels in Liver and Kidney Tissue

Tissue concentrations of CRP, TNF-α, IL-1β, and IL-6 were determined using commercial ELISA kits according to the manufacturer’s protocols. Optical density readings were obtained at 450 nm using a microplate reader [28].

### 2.8. Histopathological Examination

Collected liver and kidney tissues were fixed in 10% neutral buffered formalin, processed routinely, and embedded in paraffin. Sections (5 µm) were stained with hematoxylin–eosin (H&E) and examined using a light microscope (E-400; Nikon Corp., Tokyo, Japan) equipped with a DS-Ri2 video camera (DS-U3; Nikon Corp.).

Histopathological alterations including hepatocellular degeneration, necrosis, and sinusoidal dilatation, as well as renal tubular degeneration, tubular necrosis, and glomerular damage, were semi-quantitatively scored as negative (−), mild (+), moderate (++), or severe (+++) [26,29].

### 2.9. Statistical Analysis

Data are presented as mean ± standard deviation (SD). Statistical analyses were performed using SPSS version 21.0 (IBM Corp., Armonk, NY, USA). Differences among groups were assessed using one-way analysis of variance (ANOVA) followed by Duncan’s multiple range test for post hoc comparisons. A *p* value <0.05 was considered statistically significant.

## 3. Results

### 3.1. Hepatic Cytokine Levels

CRP levels in hepatic tissue were significantly increased in the DM and DM + Perga groups compared with the Control and Perga groups (*p* < 0.05), with no significant difference between the DM and DM + Perga groups (*p* > 0.05) (Table 1). TNF-α concentrations were significantly lower in the DM and DM + Perga groups than in the Control and Perga groups (*p* < 0.05). IL-1β levels were markedly elevated in the DM group relative to all other groups (*p* < 0.05), whereas the DM + Perga group exhibited IL-1β concentrations comparable to the Control group (*p* > 0.05). IL-6 levels were significantly higher in the DM group than in the Control and Perga groups (*p* < 0.05) but were substantially reduced in the DM + Perga group compared with the DM group (*p* < 0.05).

### 3.2. Renal Cytokine Levels

Renal CRP levels were significantly elevated in the DM group compared with the Control and Perga groups (*p* < 0.05). Although CRP concentrations were lower in the DM + Perga group than in the DM group, this reduction did not reach statistical significance (*p* > 0.05) (Table 2). The TNF-α, IL-1β, and IL-6 levels were highest in the DM group and lowest in the Perga group (*p* < 0.05). For all three cytokines, rats in the DM + Perga group exhibited significantly reduced concentrations compared with the DM group (*p* < 0.05).

### 3.3. Histopathological Evaluation

**Figure 1:**
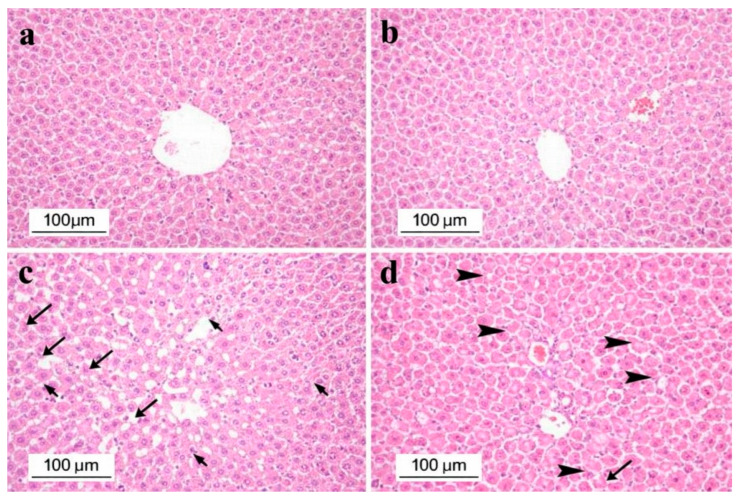
Representative H&E-stained liver tissue sections: (**a**) Control group showing normal hepatic morphology; (**b**) Perga group showing normal histoarchitecture; (**c**) DM group displaying hepatocellular degeneration, necrosis, and sinusoidal dilatation; (**d**) DM + Perga group demonstrating reduced necrosis and moderate vacuolar degeneration. Black arrows indicate areas of hepatocellular degeneration and necrosis, while arrowheads indicate vacuolar degeneration.

Histological examination of liver tissue revealed normal hepatic architecture in the Control and Perga groups. In contrast, the DM group showed marked hepatocellular degeneration and widespread necrosis, characterized by pyknotic nuclei and eosinophilic cytoplasm, accompanied by mild sinusoidal dilatation. In the DM + Perga group, necrotic areas were reduced compared with the DM group; however, hepatocellular degeneration persisted, characterized mainly by vacuolar changes (Figure 1).

**Figure 2:**
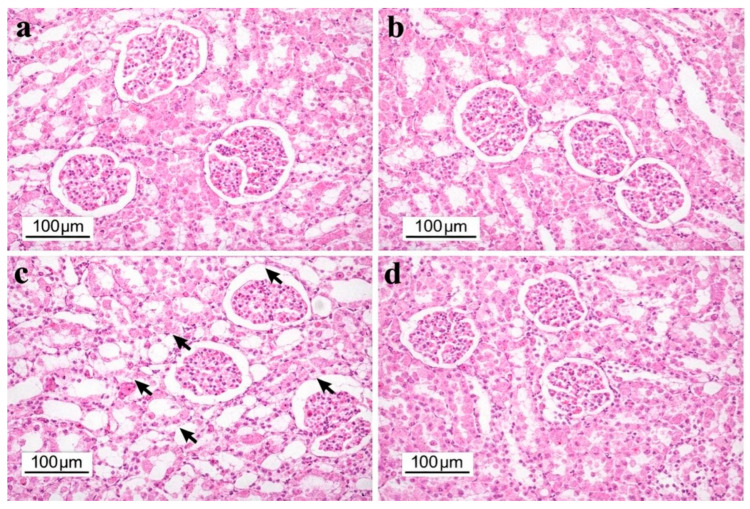
Representative H&E-stained kidney tissue sections: (**a**) Control group showing normal renal morphology; (**b**) Perga group with preserved renal architecture; (**c**) DM group displaying glomerular atrophy, widening of Bowman’s space, and tubular epithelial degeneration and necrosis; (**d**) DM + Perga group demonstrating improved renal structure with reduced degenerative changes. Black arrows indicate glomerular atrophy, widening of Bowman’s space, and tubular epithelial degeneration/necrosis.

Renal tissue architecture was preserved in both the Control and Perga groups. The DM group exhibited marked pathological alterations, including glomerular atrophy, widened Bowman’s space, and tubular epithelial degeneration and necrosis. In contrast, the DM + Perga group showed notably improved renal morphology, with structural features closely resembling those of the Control group (Figure 2).

Histopathological examination of liver and kidney tissues revealed distinct alterations among the experimental groups. In the liver, hepatocyte degeneration was absent in the Control and Perga groups, moderate (++) in the DM group, and severe (+++) in the DM + Perga group. Hepatocyte necrosis was not observed in the Control and Perga groups but was severe (+++) in the DM group and moderate (++) in the DM + Perga group. Sinusoidal dilation was mild (+) in the DM group and absent in all other groups. In the kidney, tubular epithelial degeneration and necrosis were present only in the DM group (+) and absent in the Control, Perga, and DM + Perga groups. Glomerular damage was severe (+++) in the DM group and mild (+) in the DM + Perga group, while no glomerular abnormalities were observed in the Control and Perga groups.

Overall, these findings demonstrate that diabetes induces marked histopathological alterations in both liver and kidney tissues, whereas Perga treatment ameliorates renal injury and partially modulates hepatic damage (Table 3).

## 4. Discussion

Diabetes mellitus (DM) is a chronic metabolic disorder characterized by persistent low-grade inflammation driven by dysregulated immune responses, particularly through the activation of B and T lymphocytes and the consequent elevation of pro-inflammatory cytokines such as IL-1β and TNF-α [30]. This sustained inflammatory milieu promotes chemotactic signaling, disrupts insulin action, and contributes to the development of insulin resistance, forming the basis of metabolic dysregulation in DM. Metabolically active organs, especially the kidneys, are highly susceptible to inflammation-mediated diabetic injury, while the liver—serving as the central regulator of glucose homeostasis—experiences pronounced inflammatory stress in the context of insulin deficiency and hyperglycemia [2,31]. Therefore, modulation of tissue-specific inflammatory pathways in these organs is essential for preserving their structural and functional integrity under diabetic conditions.

In the present study, the effects of bee bread (Perga) on pro-inflammatory cytokine regulation in the liver and kidneys were comprehensively evaluated under diabetic conditions using both biochemical and histopathological approaches. The findings demonstrated that Perga significantly reduced the IL-1β and IL-6 levels in hepatic tissue and attenuated the TNF-α, IL-1β, and IL-6 levels in renal tissue compared with the untreated diabetic group. These results suggest that Perga exerts notable tissue-protective activity by modulating key inflammatory pathways implicated in the development and progression of diabetic complications.

CRP is synthesized in response to monocyte-mediated signals, primarily IL-1 and IL-6, during the acute phase of inflammation and is considered a critical inflammatory marker, particularly in type 2 DM [32,33]. Hepatocyte-derived CRP expression is tightly regulated by IL-6 and TNF-α secreted from adipocytes, thereby linking systemic inflammation to hepatic responses [33]. Elevated CRP levels have been consistently associated with an increased risk of diabetes and its vascular complications [9,34].

Hyperglycemia induces microvascular alterations that further amplify the production of inflammatory mediators, including CRP, IL-6, and TNF-α [35]. Activation of hepatic inflammatory pathways contributes not only to local hepatic dysfunction but also to systemic insulin resistance, thereby exacerbating metabolic instability in DM [36]. Under high-glucose-induced oxidative stress, hepatocytes sustain cellular damage, triggering Kupffer cell activation and subsequent release of pro-inflammatory cytokines, which disrupt liver function and promote long-term hepatic injury [37].

Prolonged hyperglycemia further activates the NF-κB signaling pathway, leading to excessive production of TNF-α, IL-1β, and IL-6 [38]. This cytokine surge contributes to characteristic histopathological alterations, including hepatocellular necrosis, pyknotic nuclei, eosinophilic cytoplasm, and granular degeneration—well-recognized hallmarks of diabetic hepatic injury [39]. Consistent with these mechanistic insights, the present study demonstrated significant increases in the CRP, IL-1β, and IL-6 levels in the DM group compared with both the Control and Perga-treated groups. Histopathological findings further revealed marked hepatic degeneration in the DM group, characterized by severe hepatocyte necrosis and cytoplasmic eosinophilia, whereas necrotic alterations were attenuated in the DM + Perga group. Nevertheless, hepatocellular degeneration persisted—and was semi-quantitatively more prominent, suggesting that Perga’s hepatic protection may be limited to the modulation of specific inflammatory pathways rather than full structural recovery. Collectively, these results indicate that the hyperglycemia-induced activation of Kupffer cells is a key driver of hepatic cytokine release and subsequent structural and functional liver damage in DM, and that Perga administration effectively attenuates these inflammatory and degenerative changes.

TNF-α is a pleiotropic cytokine with context-dependent biological functions that vary according to the receptor subtype it engages [40,41]. TNFR1, which is ubiquitously expressed, contains a cytoplasmic death domain and mediates pro-inflammatory, apoptotic, and NF-κB–driven responses [42]. In contrast, TNFR2 is restricted primarily to regulatory T cells (Tregs), endothelial cells, and certain neuronal populations, and lacking a death domain, promotes cell survival, proliferation, and anti-inflammatory signaling [6,42,43,44]. Induction and shedding of TNFR2 have been shown to suppress excessive TNF activity and support tissue homeostasis [6]. Dysregulated TNF signaling has been particularly implicated in autoimmune disorders such as type 1 DM, where functional TNF deficiency impairs Treg survival and the deletion of autoreactive CD8^+^ T cells [45]. Individuals with type 1 DM exhibit elevated basal TNFR2 expression on Tregs, and cytokine stimulation (e.g., IL-2) further enhances TNFR2 surface density while TNFR1 expression remains unchanged, shifting the balance toward the reduced availability of biologically active TNF [6,44]. This receptor bias contributes to Treg/Teffector imbalance and immune dysregulation. Consistent with this concept, Faustman et al. (2013) highlighted that selective TNFR2 agonism can restore Treg function and modulate autoreactive immune responses in type 1 DM [39].

The divergent modulation of TNF-α observed between hepatic and renal tissues suggests that inflammatory signaling in diabetes is governed by organ-specific regulatory mechanisms rather than uniform systemic amplification [3,46]. In the diabetic kidney, chronic hyperglycemia sustains NF-κB-dependent transcriptional activation in mesangial and tubular epithelial cells, thereby promoting persistent TNF-α expression and amplifying local inflammatory cascades characteristic of diabetic nephropathy [4,47]. Sustained renal TNF-α production contributes to endothelial dysfunction, oxidative stress, increased vascular permeability, and progressive structural deterioration of glomerular and tubular compartments [2,4]. This pro-inflammatory amplification reflects the well-established role of TNF-α as a central mediator of renal injury in diabetic conditions [3].

In contrast, hepatic TNF-α regulation appears to be subject to tighter immunological control, largely mediated by resident macrophages (Kupffer cells), which exhibit substantial functional plasticity and context-dependent reprogramming capacity [46,48]. TNF-α signaling through TNFR1 has been shown to drive pro-inflammatory and apoptotic pathways in hepatocytes under pathological conditions [47,49]. However, experimental models of liver regeneration demonstrate that local TNF-α production can be significantly suppressed despite the presence of inflammatory stimuli, indicating intrinsic regulatory mechanisms that limit excessive cytokine release [50,51]. Specifically, Kupffer cells isolated during regenerative phases exhibit reduced TNF-α production following endotoxin stimulation, suggesting adaptive downregulation of pro-inflammatory output to preserve hepatocellular integrity [50]. Furthermore, hepatic TNF-α mRNA expression has been reported to be undetectable during regenerative states despite preserved systemic cytokine responses, supporting the concept of localized immune modulation within the liver microenvironment [51].

Therefore, the reduction in hepatic TNF-α levels observed in our diabetic model may reflect tissue-specific adaptive modulation rather than the absence of inflammatory stress [46,50]. These findings underscore that TNF signaling in diabetes is not uniformly amplified across metabolically active organs but is instead shaped by local immune microenvironments, macrophage plasticity, and receptor-mediated regulatory networks [47,48]. Consequently, while diabetes promotes pro-inflammatory amplification in the kidney, the liver may engage compensatory mechanisms that constrain TNF-α overproduction to limit collateral hepatocellular injury [46,51].

In the present study, the reduction in hepatic TNF-α levels observed in diabetic rats, together with marked increases in IL-1β, IL-6, and CRP, may reflect a compensatory regulatory mechanism triggered by chronic inflammatory stress. This cytokine pattern may, at least in part, reflect a hypothetical shift toward preferential TNFR2-mediated signaling; however, the direct assessment of receptor-specific pathways was beyond the scope of the present study. Such a receptor bias could represent an adaptive attempt to limit hepatocellular injury by enhancing anti-inflammatory and tissue-preserving responses in the diabetic liver.

Evidence from related natural bee products supports the hepatoprotective findings of the present study. Li et al. (2017) reported that bee pollen improved insulin resistance and glucose intolerance in obese mice, thereby reducing hepatic steatosis through enhanced autophagy and activation of the AMPK/mTOR signaling pathway, which collectively decreased hepatic fat accumulation and improved insulin sensitivity [18]. Similarly, Pahlavani et al. (2020) demonstrated that propolis exerted anti-inflammatory and hepatoprotective effects by attenuating hepatocellular necrosis and downregulating the mRNA expression of pro-inflammatory cytokines in rats [19]. Consistent with these observations, Bakour et al. (2021) showed that bee bread markedly reduced aluminum-induced hepatotoxicity by lowering elevated hepatic CRP, AST, and ALT levels [13]. These studies collectively indicate that bee-derived products possess notable anti-inflammatory and hepatoprotective properties, aligning well with the hepatic improvements observed following Perga administration in the present study.

In accordance with previous findings, the present study demonstrated that Perga administration significantly decreased the IL-1β and IL-6 levels in the liver tissue of diabetic rats, indicating notable anti-inflammatory activity. However, no marked alterations were observed in the TNF-α and CRP levels compared with the untreated diabetic group. Histopathological evaluations corroborated the biochemical outcomes; the DM group exhibited pronounced hepatocyte degeneration, necrosis, and mild sinusoidal dilatation, whereas necrotic alterations were attenuated in the DM + Perga group. However, hepatocellular degeneration remained pronounced, consistent with the semi-quantitative histopathological scoring. Importantly, the degenerative pattern observed in the DM + Perga group consisted predominantly of vacuolar alterations with limited irreversible necrotic damage, suggesting metabolically reversible cellular changes rather than progressive structural destruction. Therefore, semi-quantitative severity scoring should be interpreted in conjunction with qualitative morphological characteristics. Although degenerative changes persisted, the reduction in necrosis and inflammatory cytokines indicates partial restoration of hepatic tissue integrity rather than complete structural recovery. Although its influence on TNF-α and CRP appears limited, the selective modulation of IL-1β and IL-6 suggests that Perga may preferentially target key inflammatory pathways implicated in hepatic injury under diabetic conditions.

Both experimental models and clinical studies of diabetic nephropathy have consistently demonstrated elevated circulating inflammatory mediators accompanied by immune cell infiltration in renal tissues. Upregulation of adhesion molecules and chemokines in diabetic kidneys further highlights the central role of inflammation in the pathogenesis of diabetic renal injury [52]. In diabetic rats, TNF-α expression is markedly increased in both glomeruli and renal tubules, while IL-6 mRNA levels correlate positively with the severity of mesangial expansion, a hallmark feature of diabetic kidney disease [53]. TNF-α also disrupts the balance between vasoconstriction and vasodilation by increasing endothelial permeability, thereby altering intraglomerular hemodynamics and reducing the glomerular filtration rate independently of systemic blood pressure changes [52]. In addition, TNF-α promotes excessive reactive oxygen species (ROS) production, resulting in oxidative damage to glomerular capillaries, enhanced urinary albumin excretion, and progressive renal dysfunction [27].

Elevated renal CRP, IL-1β, and IL-6 levels have been closely associated with mononuclear phagocyte-driven inflammation, which accelerates structural and functional deterioration of the diabetic kidney [53]. CRP has also been reported to exacerbate diabetic nephropathy by impairing glycemic control through mechanisms linked to insulin resistance and persistent hyperglycemia [53]. Consistent with these reports, the present study demonstrated that diabetic rats exhibited the highest renal levels of CRP, TNF-α, IL-1β, and IL-6, supporting the concept that diabetes amplifies renal inflammatory processes that contribute to progressive tissue injury and impaired renal function.

Histopathological evaluation further supported these biochemical findings, revealing severe tubular degeneration, necrosis, and glomerular injury in the DM group. Perga treatment significantly reduced the renal levels of pro-inflammatory cytokines (TNF-α, IL-6, IL-1β, and CRP), in some cases approaching values seen in the Control group. These biochemical improvements were accompanied by notable histological recovery, including diminished tubular degeneration, fewer necrotic areas, and markedly reduced glomerular damage. These results are consistent with previous studies reporting that bee bread administration alleviated tubular necrosis, attenuated chronic inflammatory infiltration, limited Bowman’s space expansion, and normalized BUN and creatinine levels in obese or toxin-exposed rat models [20,21]. Collectively, these findings reinforce the renoprotective potential of Perga, likely mediated through the modulation of diabetes-induced inflammation and oxidative stress.

Interestingly, DM elicited distinct tissue-specific inflammatory responses in the liver and kidney. In hepatic tissue, the decrease in TNF-α levels despite elevated IL-1β, IL-6, and CRP suggests the activation of an adaptive regulatory mechanism, potentially driven by preferential TNFR2 signaling with concomitant suppression of TNFR1 activity, aimed at limiting hepatocellular injury. In contrast, the marked upregulation of TNF-α, IL-1β, IL-6, and CRP in renal tissue indicates a pronounced pro-inflammatory milieu that contributes to both glomerular and tubular damage. Notably, Perga administration significantly attenuated the levels of these cytokines in both organs, underscoring its dual anti-inflammatory and cytoprotective effects. These beneficial actions are likely attributable to the bioactive constituents of bee bread, particularly flavonoids and polyphenols, which are known to modulate NF-κB signaling and reduce oxidative stress.

The selected Perga dose was determined based on previously published experimental studies demonstrating hepatoprotective and anti-inflammatory efficacy in metabolic and toxin-induced models [22,23]. This dose falls within the biologically effective range reported in the literature. Nevertheless, the absence of a dose–response design represents a limitation of the present study. Inclusion of multiple Perga doses would enable a more precise evaluation of dose-dependent effects and therapeutic range. Future investigations incorporating dose-dependent analyses are therefore warranted.

## 5. Conclusions

In conclusion, the present study demonstrates that bee bread (Perga) exerts organ-specific anti-inflammatory effects in streptozotocin-induced diabetic rats. Perga significantly attenuated renal inflammatory cytokine levels (TNF-α, IL-1β, IL-6, and CRP) and markedly improved renal histopathological alterations. In hepatic tissue, Perga selectively reduced the IL-1β and IL-6 levels and attenuated necrotic changes; however, hepatocellular degeneration persisted, indicating partial rather than complete structural recovery. These findings suggest that Perga may act as a natural modulator of cytokine-mediated inflammatory responses in diabetes, with a more pronounced protective effect in renal tissue. Further mechanistic and dose-dependent studies are required to clarify the molecular pathways underlying these tissue-specific effects and optimize its potential therapeutic application.

## Figures and Tables

**Table 1:** Effect of Perga on the CRP, TNF-α, IL-1β, and IL-6 levels in liver tissue of diabetic rats.

Group	CRP (ng/mL)	TNF-α (pg/mL)	IL-1β (pg/mL)	IL-6 (pg/mL)
**Control**	12.31 ± 1.19 ^b^	739.65 ± 135.97 ^a^	151.18 ± 27.86 ^b^	24.41 ± 3.72 ^a,b^
**DM**	14.38 ± 1.35 ^a^	556.72 ± 90.63 ^b^	184.93 ± 22.17 ^a^	28.27 ± 6.53 ^a^
**DM + Perga**	14.10 ± 0.73 ^a^	464.00 ± 102.70 ^b^	154.80 ± 13.80 ^b^	19.65 ± 2.26 ^b^
**Perga**	12.13 ± 0.42 ^b^	742.88 ± 83.87 ^a^	150.69 ± 26.76 ^b^	24.30 ± 3.96 ^a,b^

Abbreviations: CRP, C-reactive protein; TNF-α, tumor necrosis factor-alpha; IL-1β, interleukin-1 beta; IL-6, interleukin-6. Data are presented as mean ± SD. Different superscript letters within a column indicate significant differences among groups (*p* < 0.05).

**Table 2:** Effects of Perga on the CRP, TNF-α, IL-1β, and IL-6 levels in kidney tissue of diabetic rats.

Group	CRP (ng/mL)	TNF-α (pg/mL)	IL-1β (pg/mL)	IL-6 (pg/mL)
**Control**	21.42 ± 3.38 ^b,c^	1265.19 ± 327.65 ^b^	45.97 ± 10.08 ^b,c^	373.22 ± 114.09 ^b^
**DM**	31.24 ± 3.50 ^a^	1970.50 ± 357.16 ^a^	89.53 ± 17.67 ^a^	616.47 ± 130.12 ^a^
**DM + Perga**	27.09 ± 3.38 ^a,b^	1343.18 ± 288.38 ^b^	56.09 ± 15.44 ^b^	457.74 ± 133.52 ^b^
**Perga**	16.61 ± 5.23 ^c^	829.56 ± 267.70 ^c^	35.19 ± 5.9 ^c^	226.36 ± 65.47 ^c^

Abbreviations: CRP, C-reactive protein; TNF-α, tumor necrosis factor-alpha; IL-1β, interleukin-1 beta; IL-6, interleukin-6. Data are presented as mean ± SD. Different superscript letters within a column indicate significant differences among groups (*p* < 0.05).

**Table 3:** Semi-quantitative histopathological scoring of liver and kidney lesions across groups *.

Tissue	Changes/Lesions	Control	DM	DM + Perga	Perga
**Liver**	Degeneration of hepatocytes	−	**++**	**+++**	−
	Necrosis of hepatocytes	−	**+++**	**++**	−
	Sinusoidal dilatation	−	**+**	−	−
**Kidney**	Degeneration of the tubular epithelium	−	**+**	−	−
	Necrosis of the tubular epithelium	−	**+**	−	−
	Glomerular damage	−	**+++**	**+**	−

* Lesions were scored as negative (−), mild (+), moderate (++), or severe (+++).

## Data Availability

The datasets generated and analyzed during the current study are available from the corresponding author on reasonable request. All biochemical data and histopathological records are securely stored and can be shared for academic and non-commercial research purposes.

## Abbreviations

ALT	Alanine aminotransferase
AST	Aspartate aminotransferase
CRP	C-reactive protein
DM	Diabetes mellitus
ELISA	Enzyme-linked immunosorbent assay
H&E	Hematoxylin and eosin
IL-1β	Interleukin-1 beta
IL-6	Interleukin-6
NF-κB	Nuclear factor kappa B
ROS	Reactive oxygen species
STZ	Streptozotocin
TNF-α	Tumor necrosis factor-alpha
TNFR1	Tumor necrosis factor receptor 1
TNFR2	Tumor necrosis factor receptor 2
